# Protection against cholera from killed whole-cell oral cholera vaccines: a systematic review and meta-analysis

**DOI:** 10.1016/S1473-3099(17)30359-6

**Published:** 2017-10

**Authors:** Qifang Bi, Eva Ferreras, Lorenzo Pezzoli, Dominique Legros, Louise C Ivers, Kashmira Date, Firdausi Qadri, Laura Digilio, David A Sack, Mohammad Ali, Justin Lessler, Francisco J Luquero, Andrew S Azman, Oral Cholera Vaccine Working Group of The Global Task Force on Cholera Control, Philippe Cavailler, Kashmira Date, Nandini Sreenivasan, Helen Matzger, Francisco Luquero, Rebecca Grais, Lale Wiesner, Melissa Ko, Vanessa Rouzier, Corey Peak, Firdausi Qadri, Justine Landegger, Julia Lynch, Andrew Azman, David Sack, Myriam Henkens, Iza Ciglenecki, Louise Ivers, Emma Diggle, Mitchell Weiss, Alan Hinman, Kahindo Maina, Imran Mirza, Guillermo Gimeno, Myron Levine

**Affiliations:** a Department of Epidemiology, Johns Hopkins Bloomberg School of Public Health, Baltimore, MD, USA; b Environmental and Cancer Epidemiology Unit, National Centre for Epidemiology, Carlos III Institute of Health, Madrid, Spain; c Consortium for Biomedical Research in Epidemiology and Public Health (CIBERESP), Carlos III Institute of Health, Madrid, Spain; d World Health Organization, Geneva, Switzerland; e Department of Medicine, Division of Global Health Equity, Brigham and Women's Hospital, Boston, MA, USA; f Department of Global Health and Social Medicine, Harvard Medical School, Boston, MA, USA; g United States Centers for Disease Control and Prevention, Atlanta, GA, USA; h International Centre for Diarrhoeal Disease Research Bangladesh, Dhaka, Bangladesh; i International Vaccine Institute, Seoul, South Korea; j Department of International Health, Johns Hopkins Bloomberg School of Public Health, Baltimore, MD, USA; k Epicentre, Paris, France; l Médecins Sans Frontières, Geneva, Switzerland

## Abstract

**Background:**

Killed whole-cell oral cholera vaccines (kOCVs) are becoming a standard cholera control and prevention tool. However, vaccine efficacy and direct effectiveness estimates have varied, with differences in study design, location, follow-up duration, and vaccine composition posing challenges for public health decision making. We did a systematic review and meta-analysis to generate average estimates of kOCV efficacy and direct effectiveness from the available literature.

**Methods:**

For this systematic review and meta-analysis, we searched PubMed, Embase, Scopus, and the Cochrane Review Library on July 9, 2016, and ISI Web of Science on July 11, 2016, for randomised controlled trials and observational studies that reported estimates of direct protection against medically attended confirmed cholera conferred by kOCVs. We included studies published on any date in English, Spanish, French, or Chinese. We extracted from the published reports the primary efficacy and effectiveness estimates from each study and also estimates according to number of vaccine doses, duration, and age group. The main study outcome was average efficacy and direct effectiveness of two kOCV doses, which we estimated with random-effect models. This study is registered with PROSPERO, number CRD42016048232.

**Findings:**

Seven trials (with 695 patients with cholera) and six observational studies (217 patients with cholera) met the inclusion criteria, with an average two-dose efficacy of 58% (95% CI 42–69, *I*^2^=58%) and effectiveness of 76% (62–85, *I*^2^=0). Average two-dose efficacy in children younger than 5 years (30% [95% CI 15–42], *I*^2^=0%) was lower than in those 5 years or older (64% [58–70], *I*^2^=0%; p<0·0001). Two-dose efficacy estimates of kOCV were similar during the first 2 years after vaccination, with estimates of 56% (95% CI 42–66, *I*^2^=45%) in the first year and 59% (49–67, *I*^2^=0) in the second year. The efficacy reduced to 39% (13 to 57, *I*^2^=48%) in the third year, and 26% (−46 to 63, *I*^2^=74%) in the fourth year.

**Interpretation:**

Two kOCV doses provide protection against cholera for at least 3 years. One kOCV dose provides at least short-term protection, which has important implications for outbreak management. kOCVs are effective tools for cholera control.

**Funding:**

The Bill & Melinda Gates Foundation.

## Introduction

For years, cholera vaccines were used infrequently because of gaps in evidence on efficacy and field effectiveness across different populations, high costs, vaccine supply constraints, and concerns about diverting resources from other cholera-related interventions. Killed whole-cell oral cholera vaccines (kOCVs) are now becoming part of the standard cholera control and prevention toolkit, in addition to the established water, sanitation, and hygiene interventions, surveillance, and case management.^1^ Although kOCVs have been used across multiple settings and have been shown to be safe and immunogenic,^2^, ^3^, ^4^ effectiveness and efficacy studies have provided a wide range of effect estimates,^5^, ^6^, ^7^, ^8^, ^9^ hindering clear communication to policy makers and clinicians.

The current formulation of kOCVs is like those first developed in the 1970s and 1980s,^10^ and includes killed *Vibrio cholerae* whole cells from both main serotypes, Ogawa and Inaba, with the main antigen being the lipopolysaccharide of killed bacteria. The vaccines' lipopolysaccharide concentration has increased since the original vaccines were developed, and some kOCVs contain the cholera toxin B-subunit, which was shown to provide no added protection in follow-up assessments more than 6 months after vaccination.^10^ Available vaccines are licensed as two-dose regimens, although single-dose regimens have been tested and suggested as a possibility in outbreaks or when vaccine supply is low.^11^

**Research in context**

**Evidence before this study**

Killed oral cholera vaccines (kOCVs) are increasingly becoming a standard cholera prevention and control tool, although a clear synthesis of the evidence supporting the degree of vaccine-derived protection is not available. We searched PubMed and Embase electronic databases for articles in English, Spanish, French, or Chinese published before April 31, 2017, using the key words “cholera” and “vaccine” and (“efficacy” or “effectiveness” or “protect*”) in the title or abstract. We also consulted members of the WHO Global Task Force for Cholera Control Oral Cholera Vaccine Working Group for any additional publications that might have been missed by the search. We identified a variety of publications from efficacy and effectiveness studies of oral cholera vaccines. In addition, a single systematic review of oral cholera vaccines, both live and killed, was identified, which covered only early efficacy and safety trials. The results showed moderate vaccine efficacy 2 years after vaccination with two doses of kOCVs, and very scarce data from subsequent years were available. Children younger than 5 years were observed to have lower efficacy than those aged 5 years and older.

**Added value of this study**

Our study builds upon the previous review of the efficacy of kOCVs by incorporating the additional evidence (ten new manuscripts comprising eight new studies) published since 2010, including nearly all the evidence generated with the most widely used vaccine, Shanchol (Shantha Biotechnics, Hyderabad, India). In contrast to the previous review, our study incorporates field effectiveness studies that are of greater relevance to field use and includes subanalyses to help elucidate the heterogeneity in efficacy or effectiveness estimates. We found that average two-dose efficacy is similar during the first 2 years after vaccination and begins to decline in the third year with positive, but not statistically significant, protection in the fourth year. However, by contrast, one large clinical trial estimated high levels of protection in the fifth year after vaccination. Short-term effectiveness (the first year after vaccination) is similar between one-dose and two-dose regimens. Even with the inclusion of new evidence, children younger than 5 years are only about half as protected as those aged 5 years and older. Finally, we found that the median age of cases enrolled in studies had a strong positive relationship with the estimated level of protection conferred by the vaccine, which helps explain some of the differences between estimates.

**Implications of all the available evidence**

kOCVs can provide medium to high levels of protection for at least 3 years, if not longer, when provided as the standard two-dose regimen. One dose can provide similar short-term protection to two doses, making it a practical option in outbreaks in which a rapid reduction in short-term risk is needed. More research is needed to understand duration of protection of both one-dose and two-dose regimens and to understand if and when booster doses should be provided.

In 2013, a global stockpile of kOCV was created by WHO to ensure vaccine availability for cholera control in outbreaks or humanitarian crises.^12^ Gavi, The Vaccine Alliance, later committed to fund up to 70 million doses (about US$1·85 per dose) from 2014 to 2018 to expand the support for vaccination in emergency and non-emergency (hotspot) settings through the stockpile.^11^ These stockpiles, combined with the WHO prequalification of a low-cost vaccine (Shanchol; Shantha Biotechnics, Hyderabad, India) in 2011, paved the way for expanded access and increased use of the vaccine. Although travellers to cholera-prone areas commonly use kOCVs,^13^ most of the world's supply of kOCV is managed and deployed through these stockpiles. Countries wishing to use these vaccines must apply through either the emergency (International Coordinating Group) or non-emergency (Global Task Force on Cholera Control [GTFCC]) mechanisms.^1^ Supply of kOCV remains low relative to the size of the at-risk population.^1^, ^14^ The WHO prequalification of a third kOCV (Euvichol; Eubiologics, Seoul, South Korea) in 2015, led to increased availability of these vaccines, opening the possibility for larger campaigns and the broader introduction of the vaccine in high-burden areas.^15^

We present the results of a systematic review and meta-analysis of the published literature on the efficacy and effectiveness of kOCVs. Although the public health impact of kOCVs is derived from both the direct protection in vaccinated individuals and the indirect (herd) protection in both vaccinated and unvaccinated individuals, we focus this review on direct vaccine protection. We summarise the current state of evidence for kOCV protection to aid clinicians and public health decision makers to assess vaccine use at the individual and population levels.

## Methods

### Search strategy and selection criteria

This systematic review and meta-analysis adhered to the Preferred Reporting Items for Systematic Review and Meta-Analyses (PRISMA) guidelines.

We searched PubMed, Embase, Scopus, and the Cochrane Review Library databases on July 9, 2016, and ISI Web of Science on July 11, 2016, for articles containing “cholera” and “vaccine” and (“efficacy” or “effectiveness” or “protect”) in the title or abstract (appendix p 2). We imposed no restrictions on publication date or language in the initial search. We consulted GTFCC Oral Cholera Vaccine Working Group members to identify additional publications.

We defined vaccine efficacy as the relative reduction in medically attended confirmed cholera risk in individuals that received the vaccine versus those who did not, as estimated by a randomised clinical trial (RCT). We defined vaccine effectiveness as the relative reduction in risk of medically attended confirmed cholera in vaccinated compared with unvaccinated individuals as measured by a case-control, cohort, or case-cohort study. We classified pragmatic RCTs as efficacy studies because they randomly allocated the vaccine. Confirmed cholera was defined as the presence of *V cholerae* in stool or rectal swab as determined by PCR, culture, or rapid diagnostic test.

Two reviewers (QB and EF) independently assessed each abstract for inclusion in the full text review, with differences resolved by discussion and consensus. Only abstracts in English, Spanish, French, or Chinese were reviewed. Each article was categorised and flagged for full text review if they reported direct or total vaccine efficacy^16^ from an RCT or effectiveness from an observational study. Duplicates were removed before abstract review using covidence software.

### Data analysis

During the full text review, both reviewers independently extracted data from manuscripts with primary estimates of direct or total vaccine effectiveness or efficacy into an electronic database, with differences resolved by discussion and consensus. Estimates from secondary analyses of trials (eg, reanalyses presented in separate manuscripts) using alternative statistical methods or measures of protection were not extracted. We extracted relevant data from text, figures, and tables, and contacted authors when data were missing.

We extracted the primary efficacy or effectiveness estimates from each study, and all secondary estimates by number of vaccine doses, duration, and age group. The main outcomes from these meta-analyses are average efficacy and effectiveness of two kOCV doses. Given the multiple estimates, and often multiple manuscripts, from each study, we focused the primary analyses on estimates of two-dose protection reflecting the duration of the primary study endpoint. For each estimate we extracted the following data: study design, study site, inclusion criteria, vaccine type (whole-cell or whole-cell with B-subunit [WC-BS]), vaccination period, study follow-up period, method of case confirmation, efficacy or effectiveness estimate and 95% CIs, number of vaccinated or unvaccinated patients or controls, number of doses, delay between doses (if more than one), vaccine coverage, age distribution of cases, and serotype and biotype distribution of cases.

Two reviewers (QB and ASA) independently assessed the risk of bias for each study, using the Newcastle-Ottawa Scale for observational studies and the Cochrane Collaboration's tool for RCTs. We produced funnel plots with primary (two-dose) outcomes to visually assess evidence for publication bias.

We used the reported point estimates of vaccine efficacy or effectiveness and 95% CIs to calculate the standard error of each. For studies reporting one-sided CIs, we reconstructed two-sided 95% CIs using standard (asymptotic) methods.^17^ Conditional on these standard errors, we estimated the average vaccine efficacy and effectiveness separately using random effect models with an empirical Bayes estimator of the between-study variance (τ^2^) using the metafor package in R.^18^, ^19^ We assessed heterogeneity using the *I*^2^ statistic, which is interpreted as the proportion of the total variation in the estimates that is due to the heterogeneity between studies rather than sampling variance.^19^ We tested for differences between subgroups (eg, vaccine type, age group, or number of doses) by fitting linear meta-regression models with the subgroup added as a moderator, and did a Wald test for the subgroup effect estimate.^20^ Given that individual estimates of vaccine protection pertain to protection over different timeframes from vaccination, we calculated the inverse-variance weighted mean duration of each average estimate to aid the interpretation of our results.

We explored the association between the vaccine protection estimates, median age of cases, and duration of the study through visual assessment of the relationships and linear regression models with polynomial splines. For one study that reported estimates from each of the 5 years of follow-up,^6^ we used a linear regression model with the log of the median age of patients as an independent variable to estimate the association with vaccine efficacy.

This study is registered in the systematic review registry PROSPERO (2016:CRD42016048232).

### Role of the funding source

The funder of the study had no role in study design, data collection, data analysis, data interpretation, or writing of the report. The corresponding author had full access to all the data in the study and had final responsibility for the decision to submit for publication.

## Results

**Figure 1:**
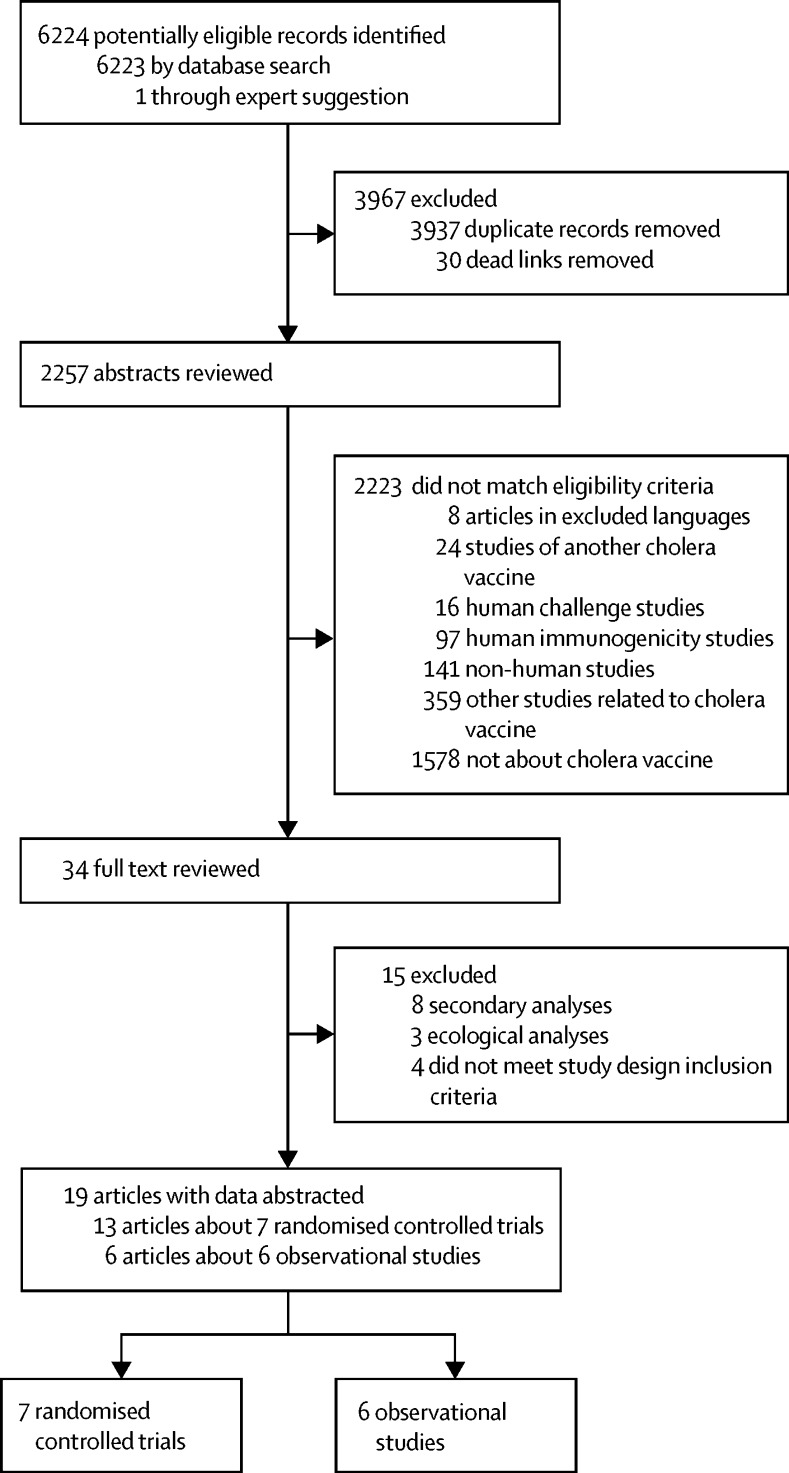
PRISMA flow chart highlighting details of the systematic review and data abstraction process Of the four studies that did not meet the study design inclusion criteria, one was excluded because it included medically attended cases and those detected from active surveillance in a clinical trial,^21^ one was excluded because of a non-standard study design,^22^ the others^23^, ^24^ only used suspected, not confirmed cholera, as the study endpoint. The 359 other studies related to cholera vaccine that did not match eligibility criteria included commentaries, reviews, policy pieces, computational modelling, and studies with non-cholera outcomes related to any cholera vaccine.

We identified 6223 records through the database search and one through consultation with experts (figure 1). 34 publications were eligible for full-text review and 19 met the inclusion criteria for data abstraction. We extracted data from seven clinical trials (13 publications^5^, ^6^, ^9^, ^10^, ^25^, ^26^, ^27^, ^28^, ^29^, ^30^, ^31^, ^32^, ^33^) and six observational studies (six publications^7^, ^8^, ^34^, ^35^, ^36^, ^37^). Two of the clinical trials were done in South America and five were in Asia; observational studies were done in Africa (four), Asia (one), and the Caribbean (one).

The seven efficacy studies included in the analyses were randomised placebo-controlled trials^5^, ^9^, ^25^, ^28^, ^29^ except for two with no placebo,^26^, ^27^ and randomisation was at the individual^5^, ^9^, ^25^, ^28^ or household and neighbourhood^6^, ^26^, ^27^ levels. In all efficacy studies, cholera was culture confirmed. Two trials included a three-dose regimen as their primary endpoint,^5^, ^9^ four used two doses^6^, ^25^, ^26^, ^27^ and one used one dose.^28^ The duration between the first two vaccine doses in trials ranged from 14 to 42 days.^6^, ^9^ Two studies had a low risk of bias across all domains of study quality,^28^, ^29^ three had a low risk of bias across most domains but at least one area with an unclear risk of bias,^5^, ^25^, ^30^ and two had at least one domain with a high risk of bias (appendix p 5).^26^, ^27^

The six effectiveness studies eligible for analysis included four case-control studies,^7^, ^8^, ^35^, ^36^ one cohort study,^34^ and one case-cohort study.^37^ Most studies enrolled all individuals seeking care for diarrhoea at study health centres as suspected cholera cases then confirmed by stool culture, except two studies that used PCR^37^ and rapid diagnostic tests^7^ in their main analyses. Controls in case-control studies included people with no diarrhoea matched spatially to patients' residences,^7^, ^8^, ^35^ and clinic-based controls with non-cholera diarrhoea.^36^ Five studies included effectiveness of a two-dose regimen as their primary endpoint,^7^, ^8^, ^34^, ^35^, ^36^ and one study used a single-dose regimen.^37^ The duration between the two primary vaccine doses in observational studies ranged from 12 to 25 days.^34^, ^35^ Three of the six studies^7^, ^8^, ^35^ had a low risk of selection bias and four^7^, ^8^, ^35^, ^37^ had a low risk of bias related to the comparability of the groups both in the design and analysis. None of the case-control studies adequately reported on response rates for both cases and controls to judge the risk of bias. The cohort and the case-cohort studies had an unclear risk of bias because of inadequate reporting on the quality of the cohort follow-up and potential differences between participants who were lost to follow-up and those who remained in the cohort (appendix p 6).

**Figure 2:**
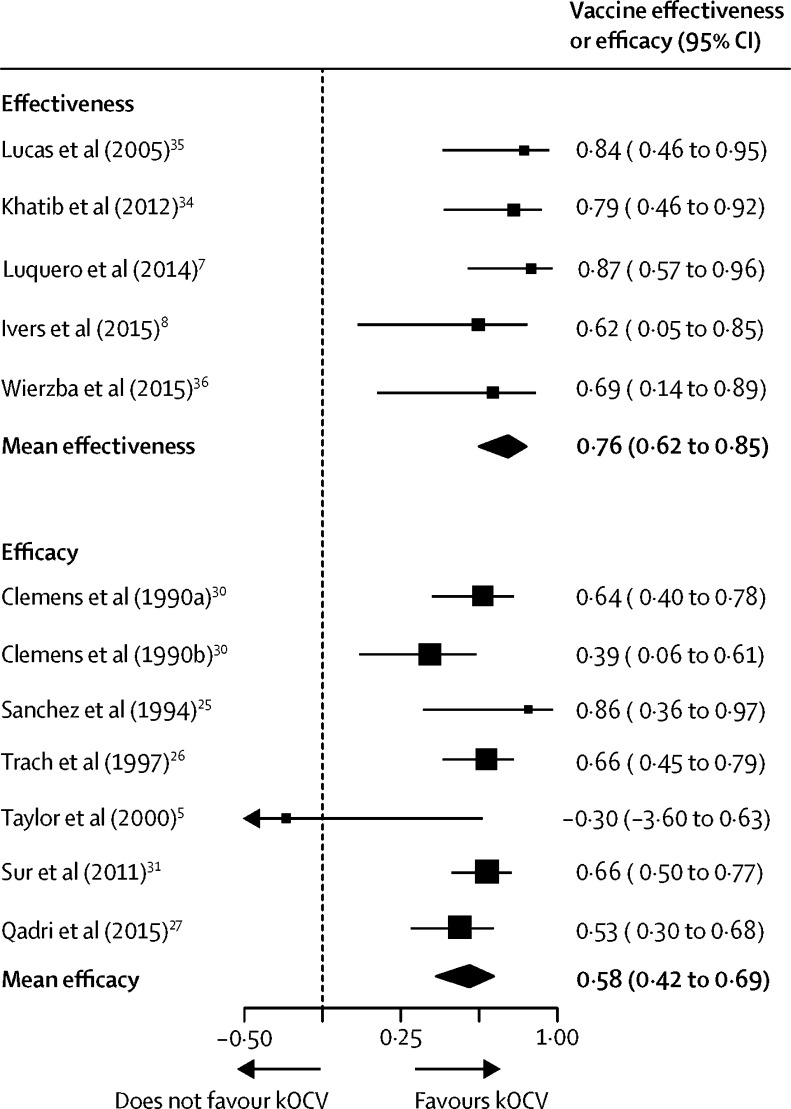
Effectiveness and efficacy main pooled analyses for two-dose killed whole-cell oral cholera vaccine 95% CIs in the figure and used for meta-analyses are not necessarily the same as those in the original study because they were based on a reconstruction of a two-sided 95% CI from estimates of the standard error of the estimate from each study. All estimates (except for those from Clemens and colleagues,^30^ 1990a and 1990b) use the main vaccine dose used in the study. Qadri and colleagues'^27^ estimate is an estimate of total protection including both direct and indirect effects. Observational study effectiveness estimates had an 18-month weighted mean duration, and the trial efficacy estimates had a 28-month weighted mean duration.

Primary estimates of two-dose regimens were available in six RCTs^5^, ^25^, ^26^, ^27^, ^30^, ^31^ and five observational studies^7^, ^8^, ^34^, ^35^, ^36^ (Table 1, Table 2). Observational study estimates pertained to protection for 5–34 months after vaccination, with an 18-month weighted mean duration (figure 2). Trial estimates pertained to protection for 4–36 months, with a 28-month weighted mean duration (figure 2). The average two-dose efficacy was 58% (95% CI 42–69, *I*^2^=58%) and the average two-dose effectiveness was 76% (62–85, *I*^2^=0). Two-dose estimates did not differ significantly by vaccine type (p=0·53, whole-cell *vs* WC-BS); however, they varied by study design (p=0·04, observational *vs* randomised designs). We did sensitivity analyses excluding trials using the WC-BS vaccines and found that the average two-dose efficacy (57·4%, weighted mean duration 28 months) and effectiveness (72·3%, weighted mean duration 22 months) were similar, although slightly lower than the those from the combined analyses. We found no signs of publication bias from a visual assessment of funnel plots (appendix p 4) for observational studies and RCTs, separately.

**Table 1:** Overview of primary efficacy studies meeting inclusion criteria in main analyses

	**Location**	**Study design**	**Vaccine**	Duration of estimate(s)*	**Dose for main outcome**	**Study population**	**Serotypes**	**Number of patients with cholera**†
Sur et al^6^, ^29^, ^31^	Kolkata, India	Cluster-randomised placebo-controlled trial	Whole-cell	2 years;^29^ 3 years (P);^31^ and 5 years^6^	Two	All non-pregnant individuals aged ≥1 year	Inaba and Ogawa	166
Taylor et al^5^	Lima, Peru	Individually randomised placebo-controlled trial	Whole-cell with B-subunit	2 years	Three	All individuals non-pregnant aged 2–65 years	Inaba and Ogawa	7
Sanchez et al^25^	Lima, Peru	Individually randomised placebo-controlled trial	Whole-cell with B-subunit	5 months	Two	Male military recruits aged 17–65 years	Not reported	16
Clemens et al^9^, ^10^, ^30^, ^32^, ^33^	Matlab, Bangladesh	Individually randomised placebo-controlled trial	Whole-cell or whole-cell with B-subunit	6 months^10^ and 1,^32^, ^33^ 3 (P),^30^ and 4^9^ years	Three	Children aged 2–15 years and all women aged >15 years, non-pregnant	Inaba and Ogawa	81 and 68‡
Qadri et al^27^§	Dhaka, Bangladesh	Cluster randomised trial	Whole-cell	2 years	Two	All non-pregnant individuals aged ≥1 year	Inaba and Ogawa	139
Qadri et al^28^	Dhaka, Bangladesh	Individually randomised placebo-controlled trial	Whole-cell	6 months	One	All non-pregnant individuals aged ≥1 year	Inaba and Ogawa	101
Trach et al^26^	Hue, Vietnam	Household randomised trials without placebo	Whole-cell	10 months	Two	All individuals aged ≥1 year	Ogawa	117

* When estimates pertaining to multiple durations (ie, times since vaccination) are presented, the primary endpoint duration that the trial design was based on is marked with (P). Some of the publications report multiple estimates with different cumulative durations, but we have included the primary estimate durations from each publication here.

† Total number of patients with cholera from both vaccination and placebo groups.

‡ Vaccine efficacy of at least one dose estimated in this study; 81 for whole-cell vaccine group and 68 for whole-cell vaccine with B-subunit group.

§ The protective estimate in the study by Qadri and colleagues^27^ was characterised as efficacy because of the cluster randomised trial design. The study had no placebo group and the non-intervention group was used as the comparison group. We consider random allocation of the exposure (vaccine) to be sufficient to classify an estimate as efficacy. The 139 patients refers to the patients in the vaccination-only intervention group that were used to assess total protection.

**Table 2:** Overview of primary effectiveness studies meeting inclusion criteria in main analyses

	**Location**	**Study design**	**Vaccine**	**Duration of estimate**	**Dose for main outcome**	**Study population**	**Serotypes**	Number of patients with cholera*
Wierzba et al^36^	Puri District, India	Case control	Whole-cell	34 months	Two	All non-pregnant individuals aged ≥1 year	Ogawa	35
Ivers et al^8^	Artibonite Department, Haiti	Case control	Whole-cell	22 months	Two	All individuals aged ≥1 year	Inaba and Ogawa	44
Luquero et al^7^	Boffa and Forecariah Districts, Guinea	Case control	Whole-cell	4 months	Two	All individuals aged >1 year	Ogawa	26†
Khatib et al^34^	Zanzibar, Tanzania	Cohort	Whole-cell with B-subunit	15 months	Two	All non-pregnant individuals aged ≥2 years	Ogawa	39
Lucas et al^35^	Beira, Mozambique	Case control	Whole-cell with B-subunit	4 months	Two	All non-pregnant individuals aged ≥2 years	Ogawa	39
Azman et al^37^	Juba, South Sudan	Case cohort	Whole-cell	2 months	One	All individuals aged ≥1 year	Inaba	34

* Total number of patients including the vaccinated and unvaccinated individuals in main two-dose analysis of study.

† Main results used were based on rapid diagnostic test results, not PCR or culture, although the results varied little by diagnostic method.

**Figure 3:**
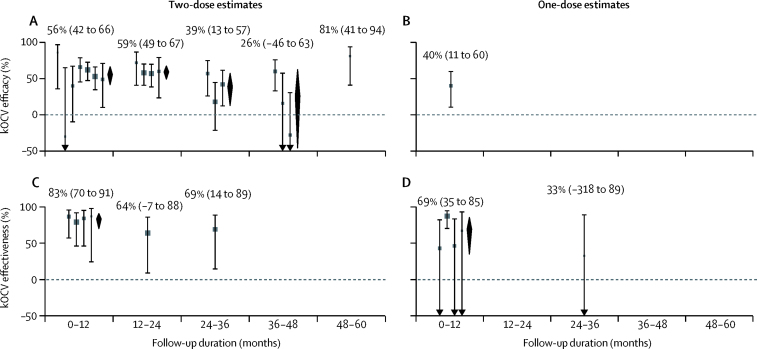
Efficacy and effectiveness by time since vaccination and dose Follow-up durations are shown here as the midpoint of the time window during which the estimate was measured. (A) Two-dose efficacy estimates at 0–12 months (from left to right, data from 25, 5, 6, 26, 9 [WC-BS group], 9 [whole-cell group], and 27), 12–24 months (6, 9 [WC-BS], 9 [whole-cell], and 27), 24–36 months (6, 9 [WC-BS], and 9 [whole-cell]), 36–48 months (6, 9 [WC-BS], 9 [whole-cell]), and 48–60 months (reference 6). The study by van Loon and colleagues^9^ has two estimates at each relevant timepoint, because results were obtained for a whole-cell vaccine group and a whole-cell with B-subunit group. (B) One-dose efficacy estimate at 0–12 months.^28^ (C) Two-dose effectiveness estimates at 0–12 months (from left to right, data from 7, 34, 35, 8), 12–24 months,^8^ and 24–36 months.^36^ (D) One-dose effectiveness estimates at 0–12 months (from left to right, data from 7, 37, 34, 8) and 24–36 months.^36^ Estimates are grouped by timeframe of analysis with zero representing the day of last dose of vaccine (dose dependent). Grey bars and squares show 95% CIs and point estimates of efficacy or effectiveness from the literature. Black diamonds show the average efficacy or effectiveness and 95% CI. Studies were grouped by time period and not all studies cover the entire 12-month period. kOCV=killed whole-cell oral cholera vaccine. WC-BS=whole-cell with B subunit.

One efficacy study^28^ and one effectiveness study^37^ used protection after one dose of kOCV as a primary outcome, both only providing estimates of short-term protection (6 months^28^ and 2 months^37^). Other studies provided estimates of one-dose protection as secondary outcomes, including four observational studies^7^, ^8^, ^34^, ^36^ and one RCT.^28^ Two studies containing one-dose efficacy estimates (or data sufficient to estimate efficacy) did not meet inclusion criteria for the outcome of medically attended confirmed cholera.^25^, ^21^ Given the paucity of evidence of longer-term single-dose protection, and the global discussions around single-dose use in outbreaks, we focused on estimating the short-term protection (up to 1 year after vaccination). The average short-term effectiveness of one-dose kOCV was 69% (95% CI 35–85, *I*^2^=62%), although this conservatively included only two estimates of cumulative effectiveness spanning over 1 year (figure 3D).^8^, ^34^ The only published one-dose clinical trial estimated 6-month efficacy of 40% (95% CI 11–60; figure 3B). Estimates did not vary significantly by study design (p=0·47, randomised trial *vs* observational). The average short-term one-dose effectiveness (69% [95% CI 35–85]; figure 3D) is similar to that of two doses (83% [79–91]; p=0·31; figure 3C), although all evidence comes from populations in which cholera transmission regularly occurs (ie, immunologically primed populations).

We identified two efficacy studies that used three kOCV doses.^5^, ^9^ One study provided three doses, each 6 weeks apart (including whole-cell and WC-BS groups).^9^ After 3 years, the results of that study showed that the efficacy was not significantly different between two and three doses (64% *vs* 50%) for the WC-BS group of the study, but two-dose efficacy was significantly lower than three-dose efficacy for the whole-cell group (39% *vs* 52%).^30^ The second study provided a third dose as a booster 10 months after the primary two-dose series and found that efficacy 2 years after the first dose was 82% (95% CI 27–95).^5^

The average efficacy estimates of kOCV were similar during the first 2 years after vaccination (figure 3; appendix p 4), with estimates of 56% (95% CI 42–66, *I*^2^=45%) in the first year and 59% (49–67, *I*^2^=0) in the second year. The efficacy reduced to 39% (95% CI 13 to 57, *I*^2^=48%) in the third year, and 26% (−46 to 63, *I*^2^=74%) in the fourth year, at which point the estimates of efficacy became highly variable between studies and the average efficacy confidence interval crossed zero. Only one study reported efficacy during the fifth year, which was 81% (95% CI 41–94).^6^

Age-group-specific estimates of protection were reported by five studies,^25^, ^26^, ^27^, ^30^, ^31^ with most dividing age groups into ages younger than 5 years, 5–15 years, and older than 15 years. We estimated the average efficacy of kOCV in children younger than 5 years to be 30% (95% CI 15–42; *I*^2^=0%, weighted mean duration of estimate 31 months), which is significantly less (p<0·0001) than in people aged 5 years or older (64%; 95% CI 58–70; *I*^2^=0%, weighted mean duration 34 months), including estimates of efficacy in people aged 15 years and older (appendix p 3). The average effectiveness in children younger than 5 years is 78% (95% CI −37 to 96, *I*^2^=0%, weighted mean duration 9 months), which is similar (p=0·77) to the effectiveness in individuals aged 5 years or older (70%, 44 to 84; *I*^2^=0%, weighted mean duration 14 months). However, the estimate for children younger than 5 years comes from only two studies containing a total of 18 patients younger than 5 years.^8^, ^35^ In school-aged children (aged 5–15 years), the average efficacy is 80% (95% CI 41–93), based on results of only two trials.^27^, ^29^

**Figure 4:**
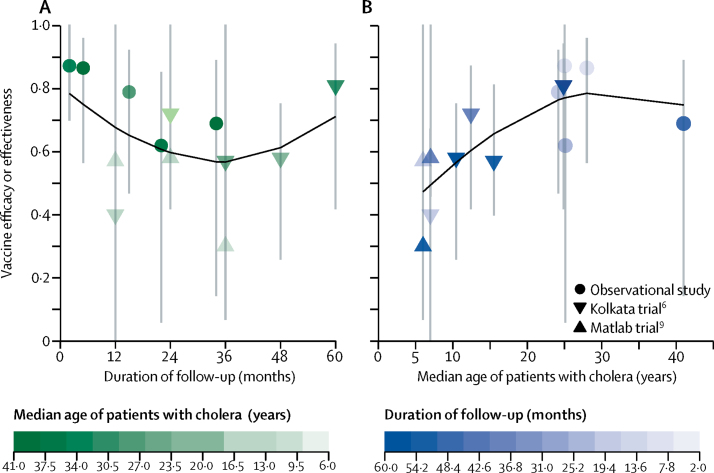
Relationship between protection by vaccine, duration of follow-up, and median age of patients with cholera (A) Relationship between protection and months of follow-up. Shading shows median age of patients with cholera. (B) Relationship between protection and the median age of patients with cholera measured in years. Shading shows duration of follow-up. Lines in black were fit with a polynomial spline with three degrees of freedom. These plots only include a subset of data in which the age distributions of patients were available (all observational studies and two trials).^6^, ^7^, ^8^, ^9^, ^34^, ^35^, ^36^ Error bars show 95% CI.

Given the differences in protection by age, we investigated whether the age distribution of cases within each study could explain the heterogeneity in efficacy or effectiveness estimates. Most studies did not report these data; however, authors of seven of the ten studies provided the requested data.^6^, ^7^, ^9^, ^27^, ^34^, ^36^, ^37^ We found that, in general, the older the patients, the higher the estimated protection (figure 4). The clearest example of this comes from the 5-year trial in Kolkata,^6^ in which a simple linear model predicts a rise of 2·0 percentage points (95% CI 0·55–3·4, adjusted *r*^2^=0·82) in efficacy for each 10% increase in the median age. Additional analyses are needed to explore this relationship between increasing age and efficacy, given that the data within studies (eg, the estimates from year to year) are correlated and that we used only a sample of data which we were able to obtain from study authors.

## Discussion

Our analyses provide a summary of the available evidence on the protection conferred by kOCVs and help clarify the observed difference in estimates. We found that kOCVs administered as the standard two-dose regimen provide a moderate to high level of protection for at least 3 years, with some evidence suggesting longer lasting protection. A one-dose regimen provides significant short-term protection, although no studies with a primary endpoint of one-dose protection after longer than 6 months have been published. Two-dose efficacy is significantly less in children younger than 5 years than in people 5 years or older.

The choice of using a one-dose or two-dose regimen is difficult, particularly during outbreaks in regions where supplies are scarce.^11^, ^38^, ^39^ Our estimates of the short-term (up to 1 year after vaccination) average one-dose and two-dose effectiveness are similar; however, this comparison was not possible for efficacy studies because of scarce data. When short-term protection is needed and two doses cannot be provided to everyone at risk, our results suggest that a single dose will provide some if not the same level of short-term protection. Most evidence for the one-dose regimen comes from observational studies. More evidence from randomised trials is needed to corroborate increasing evidence that supports the strong protection conferred by a one-dose regimen. One study compared the short-term efficacy of three doses of whole-cell and WC-BS vaccines, and concluded that the inclusion of the B-subunit might have increased short-term protection.^10^ If this finding reflects a true difference, a single-dose WC-BS vaccine could provide a more protective alternative than a whole-cell only vaccine. Whether and when to provide a booster dose remains an open question and might vary by the degree to which the population has previously been exposed to *V cholerae*. New evidence on the duration of protection by one-dose and two-dose regimens will enhance the ability to make better decisions on when each should be used and at what intervals booster doses should be provided.

kOCVs, like other oral vaccines,^40^ provide less protection to children younger than 5 years than to people aged 5 years or older. This differential protection by age might have implications for deciding between different vaccination strategies, particularly when kOCVs become more broadly used in highly endemic countries, such as Bangladesh. Vaccination of young children, despite the lower efficacy, might still have substantial effects on disease burden, because of indirect (herd) effects.^41^ More work is needed to refine estimates of the differential protection by age, to improve understanding of the effect of different age-targeted vaccine delivery strategies, and to understand whether alternative dosing regimens, such as the provision of a third dose, might enhance protection in young children.

The local epidemiology of cholera, including the pathways of transmission and the transmission intensity, can determine which age groups are at highest risk of becoming infected.^42^ In highly cholera-endemic settings such as Bangladesh and India, people with cholera tend to be younger, since older adults benefit from protection conferred by previous exposure to *V cholerae.* Given that efficacy of kOCVs is age dependent, the distribution of the age of affected people probably shapes estimates of protection from studies done in different settings (appendix pp 3–4). Frequent exposure in endemic areas can act to boost vaccine-induced immunity and prolong duration of vaccine protection, and might influence the apparent protection from the vaccine. These phenomena, in addition to the differences in the durations of the studies, might explain why the two-dose effectiveness is higher than that of two-dose efficacy (figure 2). India is the only country in which both two-dose efficacy^31^ and effectiveness^36^ studies were done, and protection estimates after about 3 years were very similar (69%^36^ and 66%^31^), showing that local epidemiology might explain apparent differences between study designs.

We identified moderate to high levels of heterogeneity between individual study estimates of vaccine protection within the primary two-dose efficacy analysis and within the average one-dose effectiveness analysis. Although some of the heterogeneity is explained by the duration of the study and the average age of patients, other unidentified factors probably also had a role. One outlier in the primary two-dose efficacy analysis, a trial done in Peru,^25^ found no protection during the 10 months after vaccination. The study^25^ assessed an early vaccine variant produced before large-scale dedicated production existed. Clemens and colleagues^43^ suggested that misclassification of outcomes within this massive household-based active surveillance programme (with twice weekly visits to households of 18 000 individuals) might have biased the results towards no effect.

This systematic review builds upon the 2011 Cochrane review^44^ of oral cholera vaccination efficacy by incorporating four new manuscripts^6^, ^27^, ^28^, ^31^ and two new clinical trials done since 2010 and including six field-effectiveness studies from a wider geographical scope, which provide measures of vaccine protection that are relevant to public health. Our estimates of two-dose efficacy and findings of low efficacy in children younger than 5 years are similar to those reported in the 2011 Cochrane review,^44^ which strengthens the rationale to look for alternative protection for young children through either herd immunity or the development of new vaccines. Additionally, we did subanalyses for protection by number of doses, age group, study type, and duration of protection to help explain some of the heterogeneity in estimates seen in the literature. This summary of the evidence is particularly timely because of the increases in global supply of kOCVs, and the increasing interest in incorporating its use into national cholera control plans in areas that have cholera outbreaks regularly.

This systematic review had multiple limitations. Given the few available studies and their diversity in study design, duration of follow-up, vaccine type, and epidemiological settings, we were unable to fully control for each factor in estimating the true average effect. We presented stratified estimates to help interpret how each factor might influence estimates of kOCV protection. In our analyses of single-dose protection, we combined estimates of one-dose protection from studies in which it was the primary outcome and studies in which it was a secondary outcome. Especially in observational settings, these secondary estimates are from individuals who did not get the full vaccine regimen, which might be correlated with cholera risk. If these data were from people who did not get the full regimen, we might have underestimated the average single-dose protection. Similarly, using both vaccine groups from the Matlab study^30^ without accounting for correlation between them (shared placebo group) led to slight underestimation of the variance in the average efficacy estimates. Last, although no significant differences between protection estimates using different vaccines were detected, the increase in antigen protection over time and the addition of the B-subunit might account for some undetected differences that this study was underpowered to detect. However, average two-dose estimates excluding the WC-BS vaccines were similar to the estimates pertaining to a combination of both vaccines.

In conclusion, kOCVs are effective in reducing the risk of cholera. Although vaccination alone will probably not lead to elimination of cholera, it can provide an important stopgap while improved water, sanitation, and health-care infrastructure are provided to vulnerable populations. More work is needed to understand how and when to best use existing vaccines and to design new and more effective ones. However, the past three decades of evidence points towards kOCV being a safe, effective, and important tool to fight cholera.
