# Supplementary material for: Protection against cholera from killed whole-cell oral cholera vaccines: a systematic review and meta-analysis
Source: Lancet Infect Dis. 2017 Oct;17(10):1080–8. doi: 10.1016/S1473-3099(17)30359-6 (PMC5639147; doi:10.1016/S1473-3099(17)30359-6)
Supplement: Supplementary appendix [file mmc1.pdf]

THE LANCET Infectious Diseases

Supplementary webappendix

This webappendix formed part of the original submission and has been peer reviewed. We post it as supplied by the authors.

Supplement to: Bi Q, Ferreras E, Pezzoli L, et al. Protection against cholera from killed whole-cell oral cholera vaccines: a systematic review and meta-analysis. *Lancet Infect Dis* 2016; published online July 17. http://dx.doi.org/10.1016/S1473-3099(17)30359-6.

Members of the Global Task Force on Cholera Control (GTFCC) Oral Cholera Vaccine (OCV) Working Group:

Agence de Médecine Préventive: Philippe Cavailler
US Centers for Disease Control: Kashmira Date, Nandini Sreenivasan
The Bill and Melinda Gates Foundation: Helen Matzger
Dipika Sur (independent)
Epicentre: Francisco Luquero, Rebecca Grais
European Commission Humanitarian Aid and Civil Protection (ECHO): Lale Wiesner
Gavi: Melissa Ko
GHESKIO: Vanessa Rouzier
Harvard University: Corey Peak
icddr,b: Firdausi Qadri
International Rescue Committee: Justine Landegger
International Vaccine Institute: Julia Lynch
Johns Hopkins University: Andrew Azman, David Sack
Médecins Sans Frontières: Myriam Henkens, Iza Ciglenecki
Partners in Health: Louise Ivers
Save the Children: Emma Diggle
Swiss Tropical and Public Health Institute: Mitchell Weiss
Task Force for Global Health: Alan Hinman
UNHCR: Kahindo Maina
UNICEF: Imran Mirza, Guillermo Gimeno (Supply Division)
University of Maryland: Myron Levine

Table S1. Specific searches and terms used in review.

Date of Query	Engine	Language Restrictions	Date Restrictions	Exact Search Query
9-July-2016	Pubmed	None	None	cholera*[Title/Abstract] AND(vaccin*[Title/Abstract]) AND (effect*[Title/Abstract] OR efficacy[Title/Abstract] OR protect*[Title/Abstract])
9-July-2016	Embase	None	None	cholera*:ab,ti AND vaccin*:ab,ti AND (efficacy:ab,ti OR effect*:ab,ti OR protect*:ab,ti)
9-July-2016	Scopus	None	None	TITLE-ABS(cholera*) AND TITLE-ABS(vaccin*) AND TITLE-ABS(efficacy OR effect* OR protect*)
11-July-2016	ISI Web of Science	None	None	TI=(cholera* AND vaccin*) AND TS=(efficacy OR effect* OR protect*)
9-July-2016	Cochrane Review Library	None	None	cholera* AND vaccin* AND (efficacy OR effect* OR protect*)

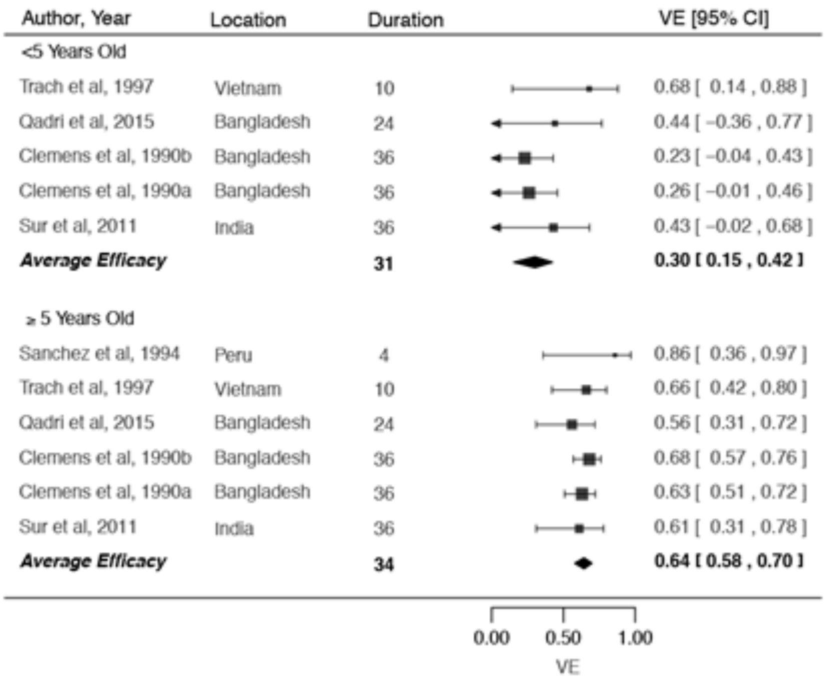

Figure S1: kOCV Efficacy by Age Group. Estimates of efficacy for under 5-year olds on the top and over 5-year olds (including estimates that only include 15 years and up, when 5 years and up not available). Light lines and bars represent estimates from the literature and their 95% confidence intervals. Diamonds represent average estimate and 95% confidence intervals.

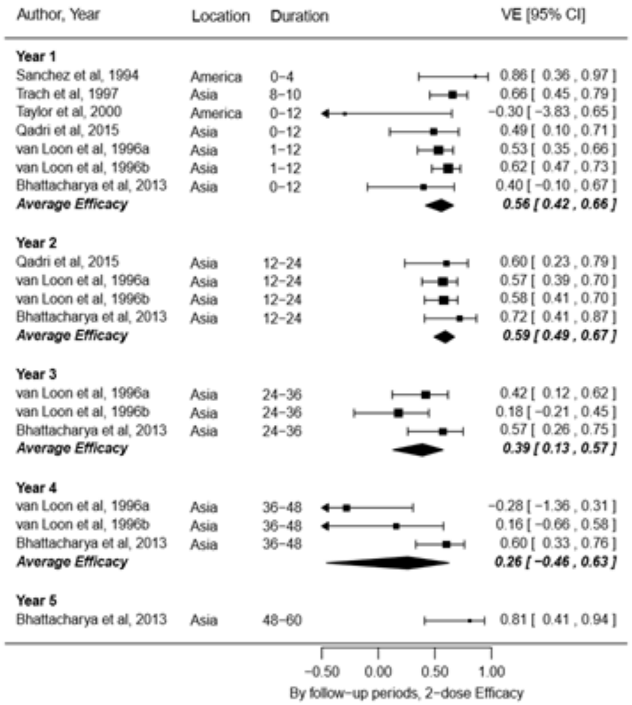

Figure S2. Two-dose vaccine efficacy by months since vaccination.

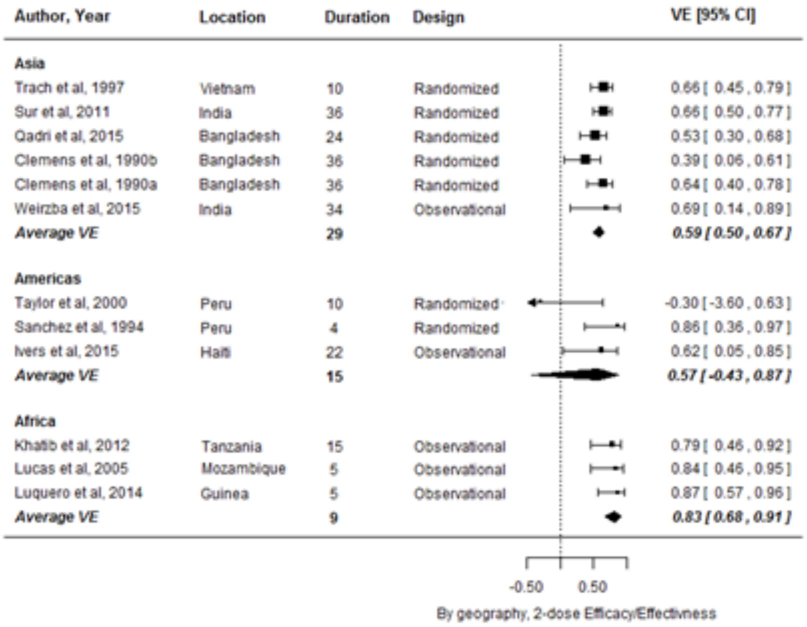

Figure S3. Two-dose pooled vaccine efficacy/effectiveness estimates by location of study.

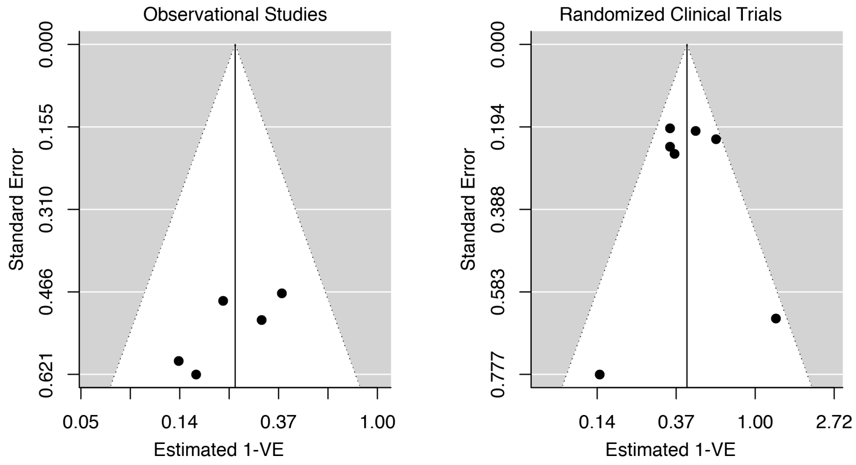

Figure S4. Funnel plot for assessing publication bias. Left-hand plot represents main two-dose estimates from observational studies and right hand plot is for randomized trials.

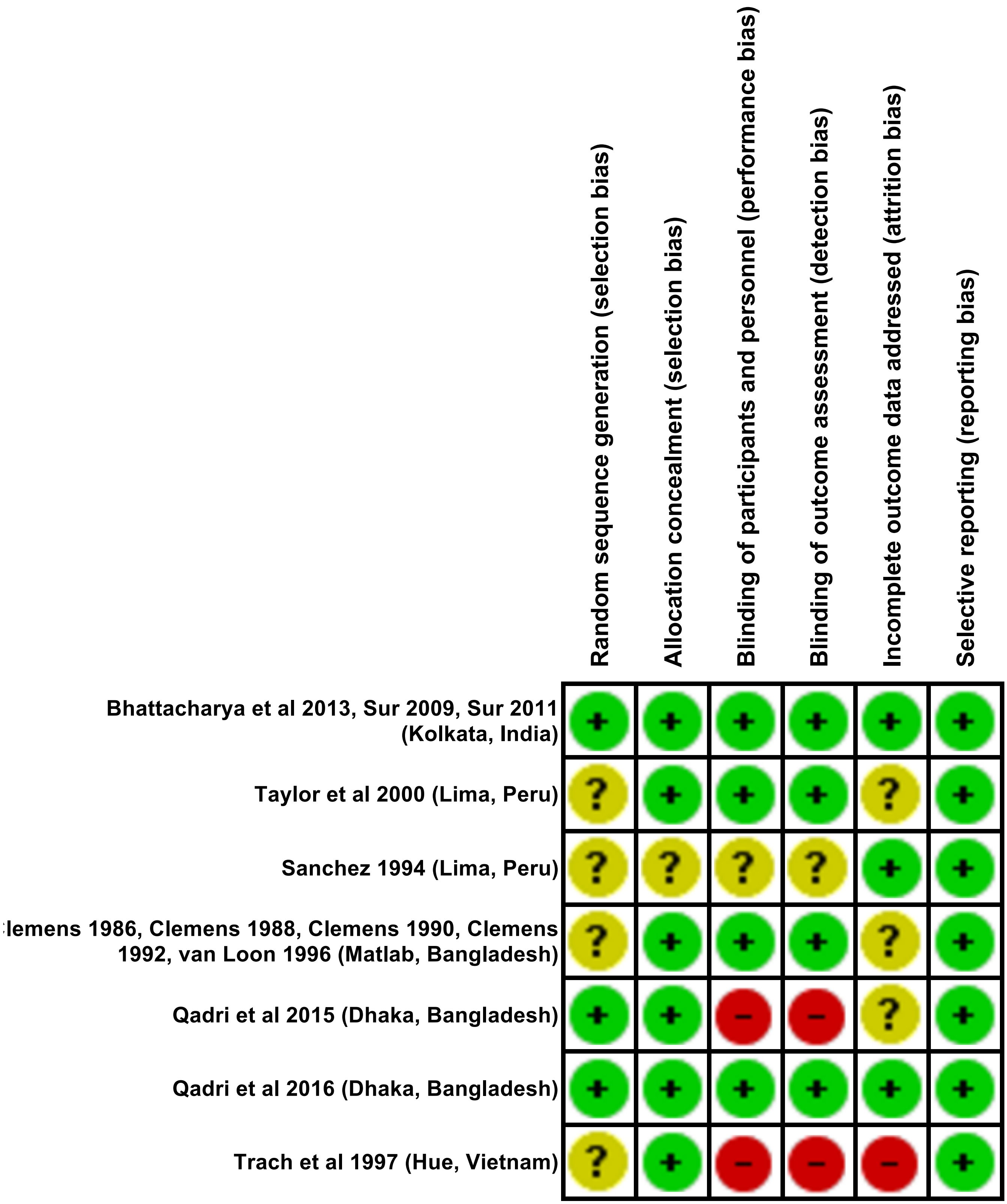

Figure S5. Risk of bias summary for clinical trials following the Cochrane Collaboration Tool. Green dots represent low risk of bias, yellow indicate unclear risk of bias and red dots indicate high risk of bias.

CASE-CONTROL STUDIES	SELECTION MAX ****	COMPARABILITY MAX **	EXPOSURE MAX ***
Lucas et al, 2005	****	**	**
Luquero et al, 2012	****	**	*
Ivers et al, 2015	****	**	**
Wierzba et al, 2015	*	*	**

COHORT STUDIES	SELECTION MAX ****	COMPARABILITY MAX **	OUTCOME MAX ***
Khatib et al, 2012	*	*	**
Azman et al, 2016	**	**	**

Figure S6. Risk of bias summary for observational studies. Note that we used the Cohort study tool to assess Azman et al, which was a case-cohort study as no tool for this study design exists. The maximum number of starts (indicating lowest risk of bias) is indicated next to MAX for each criterion.
